# Early life adversity and physical health implications in adulthood: The Multi-Ethnic Study of Atherosclerosis

**DOI:** 10.1016/j.psyneuen.2025.107674

**Published:** 2025-11-04

**Authors:** Sharon Stein Merkin, Margaret A. Sheridan, Daniela Markovic, Bonnie C. Sachs, Teresa E. Seeman

**Affiliations:** a Division of Geriatrics, David Geffen School of Medicine at UCLA, USA; b Department of Psychology and Neuroscience, University of North Carolina at Chapel Hill, USA; c Department of Medicine, David Geffen School of Medicine at UCLA, USA; d Department of Neurology, Wake Forest University School of Medicine, USA

**Keywords:** Early life adversity, Biological embedding

## Abstract

**Introduction:**

Several biological processes have been implicated in explaining the association between exposure to early life adversity (ELA) and poor health outcomes later in life, but most studies have classified ELA in terms of a simple count of adverse childhood events (ACEs). Few studies have also considered how race/ethnicity might modify these associations.

**Objectives:**

To determine to what extent different dimensions of ELA (threat and deprivation) influence cardiometabolic and inflammatory dysregulation in adulthood by race/ethnic groups.

**Methods:**

Participants in MESA, ages 45–84, were recruited from 2000 to 2002. Information regarding ELA was collected at the 2020 follow up call. Measures of inflammation (C-Reactive Protein, CRP) and cardiometabolic dysfunction (index of risk and prevalence of metabolic syndrome) were assessed at the MESA baseline exam (mid- to later life for participants); cardiovascular disease events were ascertained throughout the 20 year follow-up period. Logistic and linear regression models were fit for the relevant outcomes and adjusted for age, sex, nativity, MESA site, parental education, education, income, levels of optimism and pessimism.

**Results:**

These findings suggest some modest associations between reported experiences of ELA related to threat and deprivation and elevated CRP, metabolic dysfunction and increased risk of CVD, however these associations varied by type of ELA and by race/ethnic groups. Unexpectedly, we also found some indirect associations between reported types of ELA and lower risk of those same health outcomes.

**Conclusions:**

The inconsistent trends across race/ethnic groups, ELA dimensions and health outcomes highlight the need for a dimensional approach in classifying experiences of ELA, and suggest potential cultural differences in reporting ELA in adulthood.

## 1. Introduction

A number of biological processes have been implicated in explaining the association between exposure to early life adversity (ELA) and physical health outcomes, including inflammatory, neuroendocrine, immune, and metabolic systems (Basu et al., 2017; Su et al., 2015; Miller et al., 2011; Berens et al., 2017). In a process described by some as “biological embedding” (Miller et al., 2011; Berens et al., 2017) exposure to chronic stress can lead to dysregulation of the hypothalamic pituitary adrenal (HPA) axis (with resulting elevations in glucocorticoids) and eventually lead to altered physiology due to an overload of homeostatic responses to stress (Berens et al., 2017; Pervanidou and Chrousos, 2012). Elevated glucocorticoid levels can in turn lead to increased insulin resistance and elevated plasma glucose (Pervanidou and Chrousos, 2012; Rasgon and McEwen, 2016) excessive inflammation (Slopen et al., 2012) and, subsequently, to increased risk of cardiovascular disease (Rasgon and McEwen, 2016; Abel et al., 2012). The biological ramifications described can be particularly profound when these disruptions occur during sensitive periods of physical growth and development in childhood and adolescence (Miller et al., 2011; Pervanidou and Chrousos, 2012; Maniam et al., 2014; Liu et al., 2010).

Most of the research examining the association between exposure to ELA and adult health outcomes has operationalized ELA in one of two ways, using assessments of socioeconomic adversity or maltreatment/socioemotional adversity. Socioeconomic adversity is often measured by either parental education or occupation (Galobrades et al., 2006). Experiences of maltreatment or socioemotional adversity are generally based on prospective or retrospective reports, including socioemotional and physical measures of abuse and neglect and household dysfunction, either as individual items or combined as indicators of physical or emotional abuse (Danese et al., 2009; Suglia et al., 2014; Felitti et al., 1998; Buchmann et al., 2010 Feb; Carroll et al., 2013; Danese et al., 2007; Packard et al., 2011 Jan 17). One common practice to determine an overall measure of ELA has been to count or sum adverse events (the most well-known of these measures, perhaps, being the Adverse Childhood Events (ACEs) (Felitti et al., 1998)). Based on recent theoretical and empirical work in children, however, there is reason to consider a more dimensional approach to assessment of ELA (McLaughlin and Sheridan, 2016 Aug). McLaughlin and Sheridan developed a dimensional approach that distinguishes between two primary types of early life experiences of adversity, threat (experiences of harm or threat of harm) and deprivation (experiences characterized by the absence of expected input from a particular environment, such as parental neglect), that differentially shape learning processes early in life and long-term complex cognitive capacity (McLaughlin and Sheridan, 2016 Aug; Ellis et al., 2022 May). Using this approach to assessment of ELA, known as the Dimensional Model of Adversity and Psychopathology (DMAP), subsequent studies have largely confirmed these hypotheses, showing, for example, that early life measures of deprivation, and not threat, are associated with complex cognitive function (e.g., language, executive function), whereas threat is selectively associated with emotion dysregulation, disrupted aversive learning (Sheridan et al., 2017 Dec; Sheridan et al., 2020; McLaughlin et al., 2019; Machlin et al., 2019, 2024). Both dimensions of adversity impact long-term mental health through these different pathways, (Miller et al., 2021, 2018; Murgueitio et al., 2024) however, these dimensions have been less utilized to assess possible differential effects on cardiometabolic and inflammatory dysregulation. Considering the hypothesized biological embedding process, (Miller et al., 2011; Berens et al., 2017) where ELA exposure can lead to disease via a cascade of physiological dysregulation over time, the DMAP approach may be particularly useful in distinguishing the specific biological processes that result from different types of ELA exposure. Although a recent meta-analysis of findings on specific ELA dimensions and cardiometabolic health found individual dimensions to be less predictive of later health risk compared to cumulative measures of ELA, few studies tested differential DMAP associations in mid and late life, (Gruhn et al., nd) a time when cardiometabolic and inflammatory dysregulation may develop as a result of an accumulation of stress on these systems through young adulthood.

Aside from considering different dimensions of ELA, there is some indication that race/ethnicity may also modify the association between types of ELA and health outcomes, with evidence of different patterns of ELA experiences, as well as differential associations between ELA and health outcomes, across race/ethnic groups (Zhang and Monnat, 2021; Reynolds et al., 2024). The direction of these associations are not clear, with the possibility that race/ethnic differences in social context either exacerbate or buffer the negative health effects of early life adversity (Suglia et al., 2018). In this study, we examine whether race/ethnic identity differentially influences the association between experiences of ELA and cardiometabolic/inflammatory processes in later life.

Using the DMAP approach, we investigated whether dimensions of physical and socioemotional ELA (independent of socioeconomic adversity) differentially impact cardiometabolic and inflammatory dysregulation and CVD in mid to late life, and across diverse race/ethnic groups. Simply counting or combining various types of adversity does not allow one to identify whether distinct environmental conditions may be more or less likely to trigger the biological processes leading to disease. Moreover, development of effective interventions will benefit from a more accurate understanding of such distinct pathways if they exist, and if they differ by race/ethnic population groups. Our classification of measures of ELA into dimensions of threat (emotional/physical abuse) and markers of deprivation (lack of parental support, monitoring and household management), represents one of the first efforts to examine different aspects of ELA in a large, diverse longitudinal cohort study of adults in mid-late life. Moreover, data from the Multi-Ethnic Study of Atherosclerosis (MESA) provided a unique opportunity to examine the differential impact of ELA dimensions on multiple biological systems, as well as the cumulative impact of their dysregulation on the incidence of cardiovascular disease (CVD) across 20 years of follow-up from mid to late adulthood, and by 4 main race/ethnic groups in the United States.

### 1.1. Research Objectives

The objectives of this study were to determine whether, and to what extent, two dimensions of reported ELA (threat, and deprivation) are associated with increased risk of cardiometabolic and inflammatory dysregulation, individually and cumulatively, using an index known as allostatic load (AL), (Merkin et al., 2014 Apr) as well as incident CVD events. This study assessed the differential impact of these ELA dimensions as well as a cumulative measure of ELA, on cardiometabolic and inflammatory systems through mid to late life in 4 different race/ethnic groups in a diverse population-based longitudinal cohort study.

## 2. Methods

### 2.1. Study Sample

MESA is a prospective cohort study of the determinants of subclinical cardiovascular disease with a multi-ethnic, population-based sample of 6814 men and women with no clinical history of CVD, including white, African American, Chinese and Hispanic participants. Participants were recruited from 2000 to 2002 at ages 45–84 years from six communities in the United States, including Baltimore, MD; Chicago, IL; Forsyth County, NC; Los Angeles County, CA; New York, NY; and St. Paul, MN. The baseline examination took place between July 2000 and August 2002, with 6 follow-up exams through March 2024, as well as an annual phone follow-up, and continuous passive surveillance for CVD and mortality events. Among those screened and eligible for the baseline examination (including no history of cardiovascular disease), the participation rate was 59.8 %, and retention rates were 92 %, 89 %, 87 %, 76 %, 62 % and 58 % of the original cohort through exams 2–7, respectively. Details of the study design and recruitment for MESA have been published (Bild et al., 2002). MESA was approved by the Institutional Review Boards at participating institutions, and all participants provided written informed consent.

The study sample included participants from MESA who had available ELA data collected from telephone follow-up in year 20 (n = 3249), and data on the outcomes listed in aims above, resulting in the following study samples when excluding missing on any of the covariates: n = 3072 for inflammation, n = 3081 for metabolic syndrome, n = 3085 for cardiometabolic allostatic load, and n = 3089 for diagnosed CVD events. Most outcomes were assessed at baseline or first available exam, since the reported ELA was experienced in childhood and the baseline exam was the earliest measure available in adulthood (exceptions are noted below).

### 2.2. Exposure

ELA experiences were initially based on 7 items assessed at the MESA phone follow up 20, adapted from Felitti et al (Felitti et al., 1998). and the Risky Families questionnaire, (Taylor et al., 2004 Dec) asking participants to rate aspects of their family environment during childhood or adolescence (before age 18) on 5-point scales from one (never) to 5 (very often). Items asked whether the individual felt loved and cared for by parents, was shown physical affection from parents, was verbally abused by parent, was physically abused by parent, lived in a well-organized, well-managed household, and whether family members knew what the child was up to. There was a 7th item asking about living with a substance abuser, but both the literature and factor analysis did not support inclusion of this item as a measure of ELA (see Table 2 factor analysis). While we recognize that the non-threat related items available in MESA do not fully capture the extent of deprivation as theorized by the DMAP approach (McLaughlin and Sheridan, 2016 Aug; Machlin et al., 2019) (more commonly assessed by direct questions about parental physical and emotional neglect and lack of cognitive stimuli), we consider these measures in the current study as indicators of deprivation, distinct from measures of threat. Along with our a priori hypothesis of two distinct ELA dimensions of threat and deprivation, factor analyses suggested an additional distinction between types of deprivation. The 6 ELA items were thus classified into three dimensions: threat (emotional or physical abuse), monitoring deprivation (family not knowing what you are up to and non well-managed household) and parental warmth deprivation (not feeling loved by parent and lacking physical affection; see Table 1 for list of ELA items). Confirmatory factor analysis found goodness of fit index > 0.99 for these dimensions across race/ethnic groups. Deprivation items were reverse coded so that all ELA scores indicated greater abuse with increasing score, and the average score was calculated for each ELA dimension. In addition, we created a count index parallel to one commonly used to assess ACEs (Suglia et al., 2014) that sums binary indication of abuse (responses of at least “sometimes” for original threat questions and “never/almost never” for the original deprivation questions) across all 6 items.

### 2.3. Outcomes

Outcomes assessing inflammation and cardiometabolic dysregulation were based on data obtained at the MESA baseline exam in order assess health outcomes at the earliest age possible since the ELA exposure occurred before age 18. CVD events were obtained throughout the MESA follow-up period.

### 2.4. Inflammatory dysregulation

Inflammation was assessed using C-reactive protein (CRP; in units of milligram per liter) (Ridker et al., 2004) obtained at the MESA baseline exam. CRP values were log transformed to normalize the distribution and included in the models as a continuous measure.

### 2.5. Metabolic dysregulation

The National Cholesterol Education Program (NCEP) Adult Treatment Panel III (NCEP) criteria was used to determine metabolic syndrome at the MESA baseline exam, defined as a cluster of three or more of the following conditions: (1) waist circumference > 102 cm for men, > 88 cm for women; (2) fasting serum triglycerides > =150 mg/dL (3) high-density lipoprotein (HDL) cholesterol < 40 mg/dL for men, < 50 mg/dL for women; (4) blood pressure> = 130 mm Hg systolic or > =85 mm Hg diastolic (5) fasting blood glucose> =110 mg/dL (Grundy et al., 2004).

### 2.6. Cardiometabolic AL Score

We calculated a cardiometabolic version of allostatic load (AL; a measure of cumulative biological risk) to assess cardiometabolic risk profiles that include presymptomatic progression to disease; this score has been used in other analyses using MESA data (Merkin et al., 2014 Apr; Merkin et al., 2020). Metabolic indicators included waist–hip ratio (WHR), triglycerides, low-density lipoprotein (LDL) cholesterol, high-density lipoprotein (HDL) cholesterol, and glucose. Triglycerides, LDL cholesterol, and glucose values were considered only for those who fasted for at least 10 h. Cardiovascular measures included systolic blood pressure, resting heart rate, and pulse pressure. For each indicator, we calculated standardized scores to indicate where the individual’s value place them (in standard deviation units) relative to accepted clinical cutpoints for higher risk. These include 0.90 WHR for men and 0.85 for women, (Alberti and Zimmet, 1998) 200 milligram/deciliter for triglycerides, (National Cholesterol Education Program (NCEP) Expert Panel, 2001, 2001) 160 milligram/deciliter for LDL cholesterol, (National Cholesterol Education Program (NCEP) Expert Panel, 2001, 2001) 40 milligram/deciliter HDL cholesterol, (National Cholesterol Education Program (NCEP) Expert Panel, 2001, 2001) 4.84 log of glucose (corresponding to 126 milligram/deciliter) (Alberti and Zimmet, 1998), 140 millimeters of mercury for systolic blood pressure, (Chobanian et al., 2003) 60 millimeters of mercury for pulse pressure (Haider et al., 2003) and 90 beats per minute for heart rate (Seccareccia et al., 2001). The total AL score was calculated by summing the standardized scores for the individual parameters, and set to missing if values were missing for more than half the indicators (>4). Higher standardized scores correspond to higher risk (HDL values were multiplied by −1 since lower values reflect higher biological risk). All items for this score were based on data from the MESA baseline exam.

#### CVD Events:

Cardiovascular disease and mortality events were ascertained based on MESA participant surveillance after baseline exam through the end of December 31, 2020. Participants were contacted by telephone every 9–12 months and asked about hospital admission, CVD outpatient diagnoses, procedures and deaths. Periodic health care visits and deaths were ascertained through regular MESA study exams, participant call-ins, medical record abstractions or obituaries. Self-reported diagnoses were verified with copies of hospital/physician medical records or death certificates, and next-of-kin interviews were obtained for out of hospital CVD deaths. Two physicians independently reviewed and classified all CVD events and assigned incidence dates. In this study, we considered all CVD events according to the MESA definition, including myocardial infarction, resuscitated cardiac arrest, definite/probable angina, stroke, or deaths related to stroke, coronary heart disease or other CVD.

### 2.7. Covariates

Potential confounders included age, sex, race/ethnicity, MESA site, nativity, parental education, individual-level socioeconomic status (SES), personality measures and depressive symptoms. Race/ethnicity was classified in MESA as white, African American, Chinese and Hispanic; nativity was defined as US born vs. foreign born. Highest parental education levels were classified as less than high school, complete high school, or greater than high school education for either parent. Other SES measures included income (<$25,000, $25–49,999, and >=$50,000) and education ((<= high school, some college, and >=college), as well as wealth measures (home ownership, car ownership, land/other property ownership, investments). Personality measures were assessed using the Revised Life Orientation Test (LOT-R) (Scheier and Carver, 1985) that was administered at exam 2; pessimism and optimism were included in the models separately, as each reflect distinct personality traits (Herzberg et al., 2006) that may affect reporting bias of ELA. Depressive symptoms were assessed using the Center for Epidemiologic Studies Depression (CESD) scale as a continuous score; (Lewinsohn et al., 1997 Jun) models included CESD scores from exam 5 (proximate to when ELA data were obtained) in order to consider the influence of depressive symptoms on reporting ELA. All other covariates were obtained at MESA baseline, aside from personality and wealth measures, that were only available from the second MESA exam.

## 3. Analysis

Initially, we compared our *a priori* classification of ELA dimensions with the results of factor analysis on all 7 ELA items to test if these items statistically loaded into the same categories theoretically suggested by McLaughlin & Sheridan (i.e., threat and deprivation) (McLaughlin and Sheridan, 2016 Aug). As shown in Table 2, results of the factor analysis indicated that our proposed threat items loaded on a single factor; the proposed deprivation items loaded on two factors, one reflecting family monitoring and household organization and the other parental support and affection. In subsequent analyses we examined these distinct aspects of deprivation, and in sensitivity analyses, we considered a combined index of both sets of items in a single deprivation subscale. As expected, the single item about parental drug or alcohol use did not load on either of our two proposed dimensions and was not included in subsequent analyses of the threat and deprivation constructs.

Given the multi-ethnic nature of the MESA cohort, we also examined whether ELA reporting varied by race and ethnicity. Given the evidence for very different distributions of ELA across race/ethnic groups (described in detail in the Results), we stratified all core analyses by race/ethnicity.

Linear regression models were used to examine the association between experiences of ELA and health outcomes when considering continuous measures of CRP and cardiometabolic AL. Since CRP values were log transformed, results are interpreted as percent change in CRP; AL results are based on changes in standard deviation units from the clinical threshold. Logistic regression models were used to assess the association between ELA and prevalence of metabolic syndrome, and Cox proportional hazards models were used for incident CVD events. All models included both dimensions of threat and deprivation, with deprivation classified separately as parental warmth and parental monitoring. Separate models were also fit including the more commonly used ACE’s score. All models were adjusted for age, sex, nativity, MESA site, parental education, adult education, income, optimism and pessimism, and depressive symptoms. In order to minimize possible impacts of attrition bias (considering that experiences of ELA were only obtained at year 20 of the MESA study), all models were weighted using propensity score weights to balance the characteristics between the study and attrited samples. These propensity weights were constructed using logit regression models for attrited vs study sample participants including a variety of sociodemographic and health characteristics (age, sex, race/ethnicity, parental education, adult education, income, BMI, smoking and self-rated health). Sensitivity analyses included running unweighted models for all outcomes, as well as re-running CRP models excluding very high values corresponding to the 99th percentile (>25 milligram per liter) that may indicate infection rather than chronic inflammatory dysfunction. In addition, we ran models excluding covariates reflecting adult time-varying measures that may serve as mediators in the ELA-health associations, including depressive symptoms, optimism, pessimism, income and education.

## 4. Results

Distributions of ELA across the race/ethnic groups represented in MESA (Table 1), show some remarkable differences in ELA reporting. Most notable is the higher percentage of reported deprivation among Chinese and Hispanic participants, especially for reports of lack of physical affection from parent (42.7 % and 20.7 % respectively, compared to about 13 % for white and African American participants). Aside from lack of physical affection, Hispanic participants reported the highest levels of all other types of ELA, and either white or African American participants reported the lowest levels for almost each ELA item (aside from lowest level of physical threat among Chinese participants).

Across the outcomes assessed, we found some statistically significant associations between ELA and elevated CRP levels, as well as between ELA, metabolic syndrome and CVD events; trends in these associations varied by type of ELA, race/ethnic group, and outcome (Table 3). Reports of early life threat were associated with 16 % increased change in CRP (white participants only and increased odds of metabolic syndrome (odds ratio (OR) of 1.26, 95 % CI 1.09–1.47 for white and OR 1.44, 95 % CI 1.03–2.02 for Chinese participants). Reports of early deprivation in the form of family monitoring were associated with increased risk of CVD events (hazards ratio (HR) 1.52, 95 % CI 1.04–2.22 for Chinese participants), and deprivation in the form of lack of parental warmth was association with increased odds of metabolic syndrome (OR 1.15, 95 % CI 1.00–2.33 for African American participants). The only significant association between a higher score of ACEs and health outcomes was related to CVD events (HR 1.13, 95 % CI 1.02–1.25 for white participants only).

Unexpectedly, we also found some indication of trends opposite to what we hypothesized. Among Hispanic participants, reports of threat were associated with lower odds of metabolic syndrome (OR 0.85, 95 % CI 0.74–0.99). Assessing the interaction between ELA and nativity in this group to test for the influence of the “healthy immigrant effect” (suggested additional resilience among immigrants (Singh and Siahpush, 2002 Feb)) did not explain these findings, with similar trends for both US born and foreign-born participants (data not shown). In addition, family monitoring deprivation was associated with a 14 % decrease in CRP levels (white participants only), and parental warmth deprivation was associated with lower odds of metabolic syndrome (OR 0.82, 95 % CI 0.73–0.93 for white and OR 0.54, 95 % CI 0.40–0.72 for Chinese participants) and lower risk of CVD events (HR 0.71, 95 % CI 0.50–1.00 for Chinese participants). Assessment for differences by nativity could not be done for the other race/ethnic groups due to limited variability.

No statistically significant associations were apparent between ELA and levels of cardiometabolic AL. In sensitivity analyses, we found very similar results when re-running all unweighted models; CRP models excluding very high values also showed similar results (data not shown). Models excluding the adult time-varying measures showed similar results, aside from weaker associations between parental warmth and metabolic syndrome among white and African American participants; in both groups the trends remained but were not statistically significant.

## 5. Discussion

Our findings indicate some statistically significant association between reported ELA during childhood and greater inflammatory and cardiometabolic risk in mid-late adulthood, although we also found indication of the opposite trend. Findings were inconsistent across race/ethnic groups, health outcomes and types of ELA. While we did not find clear links between ELA and health outcomes in adulthood from this analysis, these findings provide some evidence to support a dimensional approach to examining ELA-health associations among adults in mid-late life as well as provide insight as to the distribution of ELA across race/ethnic population groups.

### 5.1. ELA and cultural diversity

The distributions of reported ELA across race/ethnic groups in the MESA population indicate that these recalled experiences may differ significantly (and in some cases, dramatically) between population subgroups. While some of the patterning of reported ELA is in line with prior work, where ELA reports are higher among minority population groups, (Lee and Chen, 2017) the MESA data reflects interesting differences in these patterns. Most notably, there is surprising similarity in ELA reporting by the white and African American participants, while Chinese and Hispanic participants reported higher prevalence (with the exception of threat levels that are lowest among the Chinese participants; see Table 1). These patterns might suggest that immigration status plays a role in explaining some of these disparities in ELA across the groups, given that 95 % of the Chinese and 63 % of the Hispanic participants are immigrants, compared to 6 % of whites and 11 % of African Americans. It is possible that 1) actual prevalence of ELA differs across ethnic/cultural background, 2) that cultural sensitivities may yield differences in reporting these experiences, or that 3) these items of ELA may not accurately reflect what is considered adversity across cultures (especially considering more subjective measures of deprivation) (Kolhatkar and Berkowitz, 2014). While some research has tested measurement invariance across similar race/ethnic groups for the emotional and physical threat measures, (Mei et al., 2022 Nov 29) few have tested the deprivation items we used in this analysis.

Measures of child neglect in particular (included in the “deprivation” dimension in the current study) lack international consensus; (Abdullah and Thattengat, 2025 Mar) these measures can differ based on cultural practices and norms, and can change over time (Kolhatkar and Berkowitz, 2014; Lansford, 2022 Apr). For example, research has found that parents demonstrate warmth differently in Asian compared to western cultures; among Asian parents, warmth is not necessarily characterized by physical affection (Lansford, 2022 Apr). This distinction might explain why our findings show a high percentage of Chinese MESA participants reporting deprivation with regard to parental warmth (43 % compared to 13–21 % in the other groups). We suggest the possibility that our inconsistent findings regarding ELA-health associations may reflect cross-cultural differences in interpretations of measures of deprivation in the diverse race/ethnic and immigrant MESA sample. Even though confirmatory factor analysis revealed general measurement invariance in terms of the psychometric properties of threat and deprivation dimension measures, these should be more rigorously tested with regard to specific health outcomes, analyses that are beyond the scope of the current study. Our findings of varied distributions of ELA by race/ethnicity and inconsistent associations with health across these groups highlight some of these issues that are crucial for future research on both measurement and health implications of ELA in diverse populations.

### 5.2. ELA Dimensions

These analyses also provide some evidence in support of utilizing a DMAP approach to classifying ELA when assessing associations with health, since trends for threat vs. deprivation dimensions were at times in opposing directions. This was especially underscored in the instances where the ACE’s score (that does not distinguish between ELA dimensions) produced null results while the separate dimensions indicated statistically significant associations in opposite directions (for example CRP and metabolic syndrome associations among white participants). Our findings suggest that different dimensions of ELA may operate differently in terms of increasing health risks in adulthood, and therefore use of the ACEs score may wash out the individual effects of ELA dimensions. Our findings were in contrast with the recent meta-analysis showing a stronger association between cumulative measures of ELA rather than dimensional measures with regard to cardiometabolic health (Gruhn et al., nd). These different results may be due to our using a limited set of adversity measures that do not adequately or consistently reflect ELA in this older sample. These findings warrant further investigation of the utility and methods of classifying ELA when assessing impact on health in mid to late life.

### 5.3. Inverse associations between ELA and health outcomes

The unexpected significant associations between ELA and negative health outcomes are also noteworthy. Despite our efforts at minimizing attrition bias, it is possible that due to attrition of those with poor health, the remaining cohort reflects individuals who are especially resilient despite experiences of adversity in early life. In a recent paper outlining a comprehensive framework for research on resilience in the aftermath of childhood adversity, Pasteuning et al. describe the dynamic trajectories of resilience that can include stable, deteriorating and also positive functioning in terms of mental and physical health changes throughout the lifecourse, (Pasteuning et al., 2024 Apr 18) Research has indeed shown some evidence of experiences of post traumatic growth (PTG) following adversity, (Tedeschi and Calhoun, 1996) although little evidence has been found of PTG following childhood maltreatment; (Mohr and Lee A.., 2017) more research is needed to test this hypothesis. Other research suggests that those experiencing adversity early in life may develop coping strategies over the lifecourse that moderate health outcomes over time and may explain these findings based on a mid-late adult cohort (Misiak et al., 2022). It is also possible that some of the cultural interpretations of the ELA items may have contributed to these unexpected findings.

### 5.4. Strengths and limitations

This study is one of the few to examine whether experiences of ELA are associated with a variety of physical health outcomes in a diverse population-based sample and to take account of different dimensions of ELA in relation to various physical health outcomes. There are, however, a number of limitations to our analyses that should be acknowledged. MESA participants were only asked about experiences of ELA in year 20 of the follow-up, when participants were ages 64 and older. While other studies have shown that retrospective accounts of ELA are associated with negative health outcomes and that recall bias can be minimized when considering objective health outcomes, (Reuben et al., 2016) the possibility of recall bias remains. Also, although we used propensity scores to minimize potential bias due to attrition from baseline to the follow-up call at year 20, the possibility remains that those who remained in the cohort through year 20 and thus were able to provide ELA information represent a biased subsample. In addition, there is limited information about early childhood conditions (aside from parental education, included in the models) that may confound the relationship between reported ELA and later health effects. Moreover, considering that the biological impact of ELA may take place during early sensitive periods of childhood and adolescence, (Miller et al., 2011; Pervanidou and Chrousos, 2012; Maniam et al., 2014; Liu et al., 2010) MESA data are limited in distinguishing those time points that may have differential impact on health outcomes, and thus may have contributed to our inconsistent findings. It is also possible that our findings suggest that the health impact of such early life experiences weaken with time or intervening life events, and that cardiometabolic risk related to experiences of ELA can be better identified more proximate to when these experiences occur. Finally, the limited ELA items in MESA may not fully capture the DMAP construct, with only limited measures of threat and deprivation, and thus might lead to under-estimation of the differential effects of ELA type and health outcomes.

## Conclusion

These findings provide some evidence for association between reported experiences of ELA in childhood and increased inflammation and cardiometabolic risk during adulthood, as well as evidence that this association may differ across race/ethnic groups and by type of ELA. These findings do not provide robust evidence for association between ELA and physical health outcomes but suggest that measures of ELA may be culturally sensitive and that studies may require more time sensitive data to better capture health effects more proximate to when ELA occurred.

## Supplementary Material

1

## Figures and Tables

**Table 1:** Select Characteristics of MESA Analytic Sample n = 3249.

Select Characteristics	Race/Ethnicity			

	White	African American	Chinese	Hispanic
	n = 1361	n = 838	n = 364	n = 686
**Age** (mean; median; std)	58.4; 57.0; 8.7	58.4; 57.0; 8.5	58.0; 57.0; 8.5	57.1; 56.0; 8.4
**Male** %	45.6	41.3	48.1	46.9
Early Life Adversity Items* %				
**Deprivation (monitoring)**				
Family did not know what you were up to	5.2	5.6	10.3	13.7
**Deprivation (monitoring)**				
Non well-managed/organized household	3.4	2.3	5.0	6.3
**Deprivation (parental warmth)**				
Parent did not make you feel loved, supported	5.4	4.8	9.1	11.2
**Deprivation (parental warmth)**				
Lack of physical affection from parent	12.8	13.1	42.7	20.7
**Threat (emotional)**				
Parent swore at, insulted, put up down	14.3	11.2	6.9	18.8
**Threat (physical)**				
Parent pushed, grabbed, shoved or hit	5.3	8.7	4.7	15.4
**ELA Dimensions** (mean; median; std)				
Threat	1.4; 1.0; 0.7	1.3; 1.0; 0.7	1.2; 1.0; 0.6	1.6; 1.0; 0.9
Deprivation- monitoring	1.6; 1.5; 0.7	1.5; 1.5; 0.7	2.1; 2.0; 0.8	1.8; 1.5; 1.0
Deprivation- parental warmth	1.9; 2.0; 0.9	1.9; 1.5; 0.9	2.6; 2.5; 1.1	2.1; 2.0; 1.1
ELA Total Count (ACEs)** (mean; median; std)	0.5; 0; 0.9	0.5; 0; 0.9	0.8; 0.5; 1.1	0.7; 0; 1.4
**Health Outcomes** ***				
**CRP** mg/L (mean; median; std)	3.1; 1.6; 4.3	4.6; 2.5; 6.5	2.0; 0.9; 4.4	4.0; 2.4; 5.3
**Metabolic Syndrome** %	28.7	32.1	22.3	40.2
**Allostatic Load** (mean; median; std)	−8.9; −8.7; 3.7	−8.4; −8.4; 3.6	−8.3; −8.3; 3.4	−7.1; −7.0; 3.7
**CVD** %	13.0	11.2	9.9	14.1

MESA: Multi-Ethnic Study of Atherosclerosis; ELA: early life adversity; ACEs: adverse childhood events; CVD: cardiovascular disease

* Response: Never, almost never, sometimes, fairly often, very often (% in table indicates >=sometimes for threat; never/almost never for deprivation)

** Sum of binary items (range 0–6)

*** Sample sizes vary: for CRP (whites n = 1353; AA n = 830; Chinese n = 363; Hispanic n = 683), for Allostatic Load (whites n = 1357; AA n = 837; Chinese n = 364; Hispanic n = 684), for Metabolic Syndrome (whites n = 1355; AA n = 836; Chinese n = 363; Hispanic n = 685), and for CVD (whites n = 1360; AA n = 838; Chinese n = 364; Hispanic n = 686).

**Table 2:** Factor Analysis of Early Life Adversity (ELA) Items.

ELSPA Items	Rotated Factor Pattern*			

	Factor1	Factor2	Factor3	Factor4
**Threat (emotional)** Parent swore at, insulted, put up down	***0.67664***	−0.13103	−0.20487	0.29873
**Threat (physical)** Parent pushed, grabbed, shoved or hit	***0.73206***	−0.14441	−0.13334	0.15357
**Deprivation (monitoring)** Family did not know what you were up to	−0.08205	***0.58640***	0.21274	−0.09180
**Deprivation (parental warmth)** Parent did not make you feel loved, supported	−0.26826	0.40223	***0.54610***	−0.08788
**Deprivation (parental warmth)** Lack of physical affection from parent	−0.13011	0.26553	***0.66192***	−0.02068
**Deprivation (monitoring)** Non well-managed/organized household	−0.14074	***0.57553***	0.21374	−0.10844
Lived with substance abuser (alcohol, drugs)	0.24990	−0.12277	−0.02765	***0.52353***

* Based on all available ELA data for MESA participants

**Table 3:** Early Life Adversity and Biomarkers/Health Outcomes.

Health Outcome*	White	African American	Chinese	Hispanic

CRP (natural log) at Exam 1^a^	Regression Coefficients (95 % Confidence Intervals)			
	n = 1298	n = 779	n = 348	n = 647
**Threat**	0.16** **(0.06, 0.26)**	−0.02 (−0.15, 0.12)	−0.04 (−0.24, 0.16)	−0.04 (−0.13, 0.06)
**Deprivation- monitoring**	−0.14** **(−0.24, −0.04)**	−0.04 (−0.18, 0.09)	0.07 (−0.10, 0.23)	0.01 (−0.10, 0.11)
**Deprivation- warmth**	−0.02 (−0.10, 0.06)	0.03 (−0.07, 0.13)	0.06 −0.08, 0.20)	0.08 (−0.02, 0.17)
**ACEs**	−0.005 (−0.07, 0.06)	−0.04 (−0.14, 0.06)	0.06 (−0.05, 0.17)	0.03 (—0.03, 0.08)
Metabolic Syndrome at Exam 1^a^	Odds Ratio (95 % Confidence Intervals)			
	n = 1300	n = 784	n = 348	n = 649
**Threat**	1.26** **(1.09, 1.47)**	0.90 (0.74, 1.09)	1.44* **(1.03, 2.02)**	0.85* **(0.74, 0.99)**
**Deprivation- monitoring**	0.95 (0.81, 1.10)	0.96 (0.79, 1.16)	1.33~ (0.99, 1.78)	1.08 (0.93, 1.26)
**Deprivation- warmth**	0.82** **(0.73, 0.93)**	1.15* **(1.00, 1.33)**	0.54*** **(0.40, 0.72)**	0.97 (0.84, 1.11)
**ACEs**	1.05 (0.95, 1.16)	1.01 (0.88, 1.16)	1.02 (0.85, 1.24)	0.96 (0.89, 1.05)
	Regression Coefficients (95 % Confidence Intervals)			
Allostatic Load at Exam 1^a^	n = 1303	n = 785	n = 349	n = 648
**Threat**	0.11 (−0.20, 0.43)	−0.20 (−0.61, 0.22)	−0.18 (−0.83, 0.47)	−0.20 (−0.52, 0.12)
**Deprivation- monitoring**	−0.24 (—0.54, 0.06)	−0.38~ (−0.80, 0.05)	0.12 (−0.42, 0.66)	0.23 (−0.12, 0.57)
**Deprivation- warmth**	−0.15 (−0.39, 0.09)	0.29~ (−0.03, 0.60)	−0.10 (−0.57, 0.36)	0.003 (−0.31, 0.31)
**ACEs**	−0.04 (−0.24, 0.17)	0.01 (−0.30, 0.32)	0.07 (−0.29, 0.42)	−0.03 (−0.22, 0.16)
	Hazard Ratio (95 % Confidence Intervals)			
Cardiovascular Disease Events (Exam 1-December 31, 2020)^a^	n = 1304 171 events	n = 786 87 events	n = 349 35 events	n = 650 96 events
**Threat**	0.89 (0.73, 1.08)	1.13 (0.89, 1.45)	0.76 (0.45, 1.30)	1.08 (0.90, 1.29)
**Deprivation- monitoring**	1.10 (0.93, 1.29)	0.84 (0.64, 1.09)	1.52* **(1.04, 2.22)**	1.03 (0.85, 1.24)
**Deprivation- warmth**	1.07 (0.94, 1.22)	1.18 (0.98, 1.42)~	0.71* **(0.50, 1.00)**	0.93 (0.78, 1.10)
**ACEs**	1.13* **(1.02, 1.25)**	1.02 (0.85, 1.22)	0.96 (0.75, 1.24)	0.99 (0.88, 1.10)

~ 0.1 <p < =0.05;

* 0.01 <p < 0.05;

** 0.001 <p < 0.01;

*** p < 0.001

a All models adjusted for age, sex, nativity, MESA site, parental education, adult education income; optimism, pessimism, and depressive symptoms at exam 5. CRP:C-Reactive Protein

## Appendix A. Supporting information

Supplementary data associated with this article can be found in the online version at doi:10.1016/j.psyneuen.2025.107674.
