# Supplementary material for: Early life adversity and physical health implications in adulthood: The Multi-Ethnic Study of Atherosclerosis
Source: Psychoneuroendocrinology. Author manuscript; Available in PMC 2026 Aug 25. (PMC13505205; doi:10.1016/j.psyneuen.2025.107674)
Supplement: Supplementary file 1 [file NIHMS2185875-supplement-1.docx]

**Table S1: Covariates for MESA Analytic Sample n=3249 ***

| **Select Characteristics** | **Race/Ethnicity** | | | |
| --- | --- | --- | --- | --- |
|  | **White**  n=1361 | **African American**  n=838 | **Chinese**  n=364 | **Hispanic**  n=686 |
| **Nativity** (% Born Abroad) | 6.0 | 10.6 | 95.3 | 63.7 |
| **MESA Site** % |  |  |  |  |
| Forsyth County, North Carolina | 19.9 | 29.6 | 0 | 0.3 |
| Newy York, New York | 8.2 | 22.1 | 0.3 | 34.6 |
| Baltimore, Maryland | 18.8 | 25.7 | 0 | 0 |
| St. Paul, Minnesota | 27.2 | 0 | 0 | 37.6 |
| Chicago, Illinois | 20.3 | 14.1 | 48.4 | 0 |
| Los Angeles, California | 5.6 | 8.6 | 51.4 | 27.6 |
| **Parental Education** % |  |  |  |  |
| Less than High School | 23.0 | 40.5 | 43.5 | 69.6 |
| Completed High School | 31.7 | 31.8 | 22.6 | 21.4 |
| High School or More | 45.4 | 27.8 | 33.9 | 9.1 |
| **Education** % |  |  |  |  |
| <=High School | 17.1 | 24.7 | 33.5 | 56.0 |
| Some College | 27.4 | 37.7 | 21.7 | 30.6 |
| College or More | 55.4 | 37.6 | 44.8 | 13.4 |
| **Income** % |  |  |  |  |
| <$25,000 | 10.1 | 21.0 | 38.4 | 37.1 |
| $25-49,999 | 26.4 | 33.8 | 24.3 | 39.1 |
| >=$50,000 | 63.6 | 45.2 | 37.3 | 23.9 |
| **Optimism** (mean; median; std) | 9.7; 10.0; 2.0 | 10.3; 11.0; 1.8 | 10.0; 10.0; 2.0 | 10.1; 11.0; 2.1 |
| **Pessimism** (mean; median; std) | 4.7; 4.0; 2.0 | 5.0; 4.0; 2.2 | 5.8; 6.0; 2.3 | 5.2; 5.0; 2.3 |
| **CESD Score** (mean; median; std)* | 7.6; 6.0; 7.2 | 7.6; 6.0; 7.4 | 7.5; 6.0; 7.0 | 9.2; 7.0; 8.4 |

MESA: Multi-Ethnic Study of Atherosclerosis

CESD: Center for Epidemiologic Studies Depression

*Sample sizes smaller for some of the covariates due to missing values.

CESD: n=1249 for white, n=749 for AA, n=332 for Chinese and n=619 for Hispanic participants; Income: n=1332 for white, n=796 for AA, n=362 for Chinese and n=671 for Hispanic participants; Optimism and pessimism: n=1324 for white, n=809/808 for AA, n=355 for Chinese and n=661 for Hispanic participants;

Parental education: n=1324 for white, n=806 for AA, n=354 for Chinese and n=660 for Hispanic participants; Education and nativity: n=1360 for white, n=833 for AA participants.
